# Comparative Analysis of Davidson and Glyoxal Fixatives on Autofluorescence and Immunolabeling in Medaka (*Oryzias latipes*) Tissues

**DOI:** 10.3390/biomedicines13082002

**Published:** 2025-08-18

**Authors:** Li Xiao, Eriko Sato, Ryoji Ide, Naohiro Shimamura, Chikako Saiki, Nobuhiko Miwa

**Affiliations:** 1 Department of Physiology, School of Life Dentistry at Tokyo, The Nippon Dental University, 1-9-20 Fujimi, Chiyoda-ku, Tokyo 102-8159, Japan; e-sato2124013@tky.ndu.ac.jp (E.S.); ryo-ide@tky.ndu.ac.jp (R.I.); chikako@tky.ndu.ac.jp (C.S.); 2 Department of Dental Anesthesiology, School of Life Dentistry at Tokyo, The Nippon Dental University, 1-9-20 Fujimi, Chiyoda-ku, Tokyo 102-8159, Japan; shimamura@tky.ndu.ac.jp; 3 Incorporated Association Hydrogen Medical Institute, Minatojima Minamicho 1-6-4, ChuOh-Ku, Kobe 650-0047, Japan; vitamin2002rejuvenation@yahoo.co.jp; 4 Faculty of Life Sciences, Prefectural University of Hiroshima, Hiroshima 727-0023, Japan

**Keywords:** medaka (*Oryzias latipes*), brain, neuronal markers, fixation, glyoxal, Davidson’s solution, autofluorescence, imaging, histology

## Abstract

**Background**: Fixation influences the quality of staining across species, especially in neuroscience, where accurate visualization of neuronal structures and protein localization is crucial for understanding brain function and pathology. This study compared two commonly used fixatives—9% glyoxal (G-fix) and Davidson’s solution (D-fix)—regarding their effects on autofluorescence, immunolabeling specificity, and histological quality in medaka brain tissue. **Methods:** Mixed-sex medaka from five strains were fixed with either G-fix or D-fix. Autofluorescence was assessed in posterior bodies and brain tissues, including sections stained with fluorescently conjugated secondary antibodies alone. Tissues were also injected with fluorescent dyes or immunolabeled for neuronal markers (PGP9.5, NeuN, and NCAM) using fluorescent secondary antibodies. Hematoxylin and eosin (H&E) staining and immunohistochemistry were used to evaluate tissue morphology and chromogenic antigen detection. **Results**: Both fixatives induced autofluorescence: D-fix enhanced blue signals, while G-fix increased green and red fluorescence. These autofluorescence levels were significantly weaker than those from fluorescent dyes or PGP9.5 immunolabeling. Posterior body tissue showed patterns similar to deparaffinized brain sections, supporting its use for pre-screening fixation. G-fix yielded more neuron-specific PGP9.5 staining, whereas D-fix showed broader signal distribution. NeuN and NCAM were not detected, likely due to antibody incompatibility. PGP9.5 was undetectable by immunohistochemistry, while D-fix provided superior H&E staining quality. **Conclusions**: Although both fixatives induced autofluorescence, their signals were weaker than those of conventional dyes and antibodies. Glyoxal improved specificity for neuronal immunofluorescence, while Davidson enhanced histological detail. These findings provide practical guidance for optimizing fixation strategies in medaka-based neuroscience and histopathological research.

## 1. Introduction

The medaka (*Oryzias latipes*) is a small teleost fish widely used as a model organism in developmental biology, toxicology, genetics, and neuroscience. Its small size, transparent embryos, rapid development, and well-annotated genome make it especially suitable for high-throughput and in vivo studies [1,2,3]. In recent years, medaka has also emerged as a valuable model for investigating brain development, neurodegeneration, and responses to environmental stressors due to its structural similarity to mammalian central nervous systems and its amenability to genetic manipulation [4,5]. Despite these advantages, standardization of tissue preparation methods for histology and immunostaining in medaka remains limited, particularly for fixed adult brain tissue.

Fixation profoundly influences the quality of histological and immunofluorescent staining across tissues and species, particularly in neuroscience research, where accurate visualization of fine neuronal structures and protein localization is essential for understanding brain function and pathology. In small teleost models, such as medaka and zebrafish, optimized fixation protocols are critical for obtaining reliable neuroanatomical and neuropathological data. However, different fixatives can vary substantially in their impact on antigen preservation, background autofluorescence, and compatibility with downstream staining techniques. Formaldehyde-based fixatives, such as Davidson’s solution, are widely used in fish histology due to their rapid preservation of tissue architecture and minimal induction of tissue shrinkage [6,7]. Glyoxal, a dialdehyde fixative, has recently gained attention as a non-toxic alternative to formaldehyde with improved antigen preservation and reduced protein cross-linking [8]. Recent advances in neurohistology have shown that glyoxal fixation enhances immunolabeling sensitivity for neuronal subtypes and synaptic proteins in the mouse brain, outperforming formaldehyde in multiple brain regions [9]. These findings highlight glyoxal’s promise as a neuroscience-compatible fixative and emphasize the need to assess its performance in additional model organisms like medaka. However, comparative data on glyoxal versus Davidson fixation in adult medaka brain remain scarce. 

Autofluorescence is a well-documented challenge in fluorescence-based imaging, particularly in fixed tissues. Fixation-induced autofluorescence can obscure weak or low-abundance fluorescent signals, complicating the interpretation of immunofluorescence results [10,11,12]. Its intensity and spectral properties vary depending on the fixative used and the tissue type. Although strategies to reduce autofluorescence have been explored in mammalian systems, little is known about these effects in medaka. Importantly, the accuracy of fluorescence-based neuronal imaging—especially for detecting fine neural structures or low-expression markers—relies heavily on signal clarity. Subtle shifts in the signal-to-noise ratio caused by fixation may misrepresent true protein localization, potentially leading to erroneous neuroanatomical conclusions. Thus, understanding how fixatives influence both antigen detectability and background fluorescence is essential for generating reliable and reproducible data in medaka-based neuroscience studies.

In this study, we compared glyoxal- and Davidson-fixed medaka brain tissue to evaluate their suitability for immunofluorescence (IF), immunohistochemistry (IHC), and hematoxylin and eosin (H&E) staining. Using five commercially available strains of medaka with distinct body pigmentation, we first assessed baseline and fixation-induced autofluorescence in both posterior body and brain tissues under commonly used excitation/emission channels. We then examined the detectability of neuronal markers—including PGP9.5, NeuN, and NCAM—using both IF and IHC approaches in fixed brain sections. Additionally, we evaluated the histological quality of H&E-stained brain tissue sections fixed with glyoxal or Davidson’s solution.

## 2. Materials and Methods

### 2.1. Reagents

Glyoxal (40% solution) (078-00905), acetic acid (017-00256), 99.5% ethanol (057-00456), propidium iodide (PI) (169-26281), Mayer’s Hematoxylin Solution (131-09665), and 1% Eosin Y Solution (051-06515) were purchased from FUJIFILM Wako Pure Chemical (Osaka, Japan). Davidson’s solution (16801) was obtained from Muto Pure Chemicals Co., Ltd. (Tokyo, Japan) and contained 7% formaldehyde, 2% methanol, 11.5% acetic acid, 33% ethanol, and 46.5% water. NucBlue™ Live ReadyProbes™ Reagent (Hoechst 33342, R37605) was purchased from Thermo Fisher Scientific K.K. (Tokyo, Japan). Fluoresbrite^®^ YG Carboxylate Microspheres (0.75 μm, cat. #07766-10) were purchased from Polysciences, Inc. (Warrington, PA, USA) via a local supplier.

### 2.2. Animals

Wild-type adult Japanese medaka (*Oryzias latipes*) of both sexes—including Yokihi (Yangguifei, Yang), Hi (red), blue, black, and white strains—were obtained from a local aquarium supplier. Fish were maintained in aquaria under a 12 h light/12 h dark cycle at room temperature in dechlorinated tap water (treated with 2 mL/10 L sodium thiosulfate) and fed daily with commercial dry feed. All animal procedures complied with the ARRIVE guidelines and were approved by the Animal Ethics Committee of the Nippon Dental University School of Life Dentistry at Tokyo (approval no. 24-14-1, 10 October 2024).

Experimental fish (mixed sex, 30–40 mm) were randomly assigned to each group. No significant differences in autofluorescence intensity or immunostaining patterns were observed between male and female medaka; therefore, data from both sexes were pooled for analysis.

### 2.3. Fixation

A 9% glyoxal fixative was freshly prepared by mixing 8% acetic acid with glyoxal and adjusting the pH to 4.0 using drops of 5 N NaOH, as described previously [9]. Fish were deeply sedated using cryogenic anesthesia [13], positioned on custom-designed holders fabricated with a 3D printer (ELEGOO Mars 3 Pro; ELEGOO, Shenzhen, China) (the related STL files are provided in the Supplementary Materials) and then euthanized by decapitation. The heads and posterior body were immediately fixed in either 9% glyoxal or Davidson’s solution, followed by dehydration and paraffin embedding. Brain sections (10 μm) were sliced from paraffin blocks of the fish heads using a rotary microtome (HM355S STS, Thermo Fisher Scientific). Autofluorescence in the brain sections and posterior body tissues was visualized using a laser scanning microscope (LSM700, Carl Zeiss, Tokyo, Japan) or a fluorescence stereo microscope (AxioZoom.V16, Carl Zeiss) and quantified with ImageJ software (version 1.54p, National Institutes of Health, Bethesda, MD, USA) [14].

### 2.4. Injection of Fluorescent Materials

The central region of the fixed posterior body from five medaka strains was injected with 10 µL of either 0.1% Fluoresbrite^®^ YG Carboxylate Microspheres (0.75 μm), Hoechst 33342, or PI (50 µg/mL). Fluorescence signals were observed using a fluorescence stereo microscope and quantified with ImageJ.

### 2.5. Immunofluorescence, Immunohistochemistry, and H&E Staining

Paraffin-embedded brain sections (10 μm) were used for IF, IHC, and H&E staining. Sections were deparaffinized and underwent heat-induced antigen retrieval in 0.5% ImmunoSaver solution (097-06192, FUJIFILM Wako Pure Chemical) prior to immunostaining. Primary antibodies included anti-PGP9.5 (GTX109637, GeneTex, Irvine, CA, USA), anti-NeuN (ab279296, Abcam, Tokyo, Japan), and anti-NCAM (ab133345, Abcam). Fluorescent-dye-conjugated secondary antibodies included goat anti-rabbit IgG Alexa Fluor™ 488 and Alexa Fluor™ 594 (A-11008 and A-11036, Thermo Fisher Scientific). Nuclei were counterstained with DAPI-Fluoromount-G™ (0100-20, SBA). For IHC, horseradish peroxidase (HRP)-conjugated secondary antibodies (ab6802, Abcam) and the DAB detection system [HISTOFINE SAB-PO (M) kit, Nichirei Bioscience Inc., Tokyo, Japan] were used. H&E staining was performed using a standard protocol. Imaging was carried out with a NanoZoomer S20 scanner (Hamamatsu Photonics K.K., Shizuoka, Japan) and analyzed using ImageJ.

### 2.6. Statistical Analysis

Statistical analyses were performed as described previously [15,16]. Data were analyzed using GNU PSPP Statistical Analysis Software (version 0.8.2-gad9374; https://www.gnu.org/software/pspp/, accessed on 6 March 2025) and EZAnalyze Excel-based tools (http://www.ezanalyze.com/, accessed on 6 March 2025). One-way analysis of variance (ANOVA) was conducted, followed by Tukey’s post hoc test and Bonferroni correction. A *p*-value < 0.05 was considered statistically significant. All experiments were independently repeated 3 to 5 times.

## 3. Results

### 3.1. Glyoxal- and Davidson-Fixed Medaka Tissues Exhibit Distinct Autofluorescence Profiles

**Figure 1:**
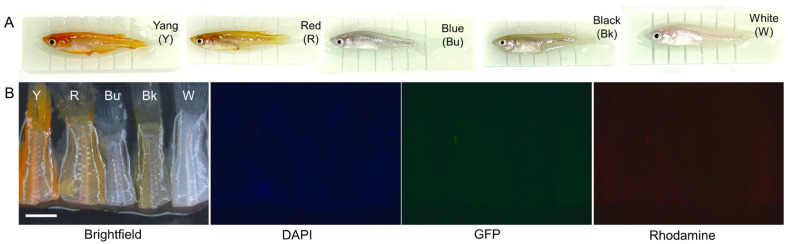
Fresh medaka tissues did not exhibit autofluorescence. Five medaka strains (n = 10 per group) were assessed for intrinsic autofluorescence. Following cryogenic anesthesia, the fish were placed on custom-designed 3D printed holders that ensured consistent positioning for decapitation. The fish were then euthanized by decapitation, and unfixed tissues were immediately examined using a fluorescence stereo microscope. (**A**) Photographs of medaka fish positioned on 3D printed holders. The long side of the holder measures 5 cm for Yangguifei (Y) and white (W) medaka and 4 cm for red (R), blue (Bu), and black (Bk) medaka. (**B**) Fluorescence images of posterior body regions under different light sources. Scale bar = 3.0 mm. Yang (Y), Yangguifei (Yokihi) medaka; Red (R), red medaka; Blue (Bu), blue medaka; Black (Bk), black medaka; White (W), white medaka. The same abbreviations are used throughout the remaining figures.

Before examining autofluorescence in fixed brain tissue, we first evaluated the background autofluorescence in the posterior bodies. Considering that body color or strain type may influence autofluorescence, we used five commonly available medaka strains—Yangguifei, red, blue, black, and white medaka—for this study. As shown in Figure 1A, these strains exhibit a range of body colors. 

Under fluorescence stereo microscopy with blue (DAPI: 357/44 nm excitation, 447/60 nm emission), green (GFP: 482/25 nm excitation, 524/24 nm emission), and red (rhodamine: 531/40 nm excitation, 593/40 nm emission) light sources, no autofluorescence was observed in the unfixed posterior body tissues of any of the five medaka strains (Figure 1B).

**Figure 2:**
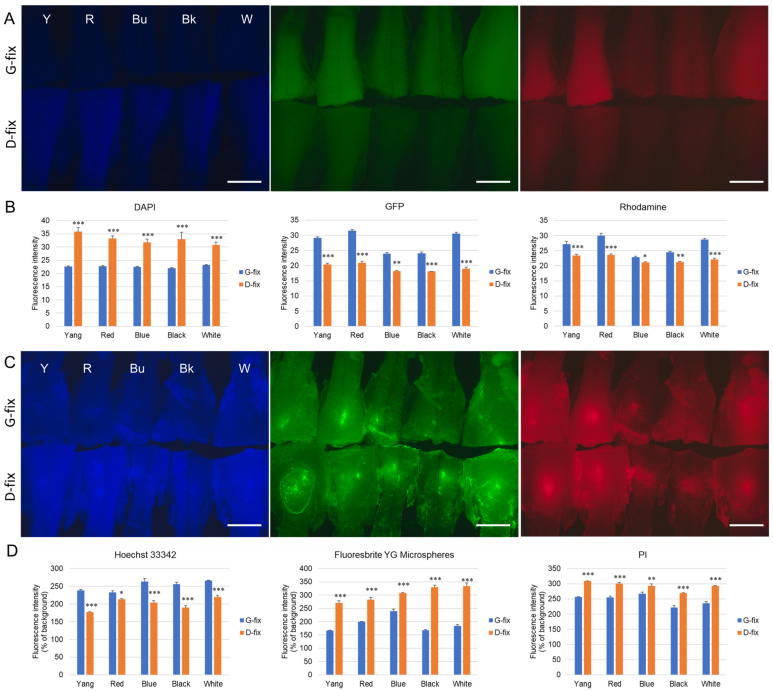
Glyoxal- and Davidson-fixed medaka tissues exhibit different levels of autofluorescence. Five types of medaka fish (n = 10 per group) were euthanized and fixed with either 9% glyoxal or Davidson’s solution, as described in the Section 2 (Materials and Methods). (**A**) Representative fluorescence images of posterior body regions under various light sources following fixation. Scale bar = 3.0 mm. G-fix: glyoxal-fixed; D-fix: Davidson-fixed. The same abbreviations are used throughout the remaining figures. (**B**) Quantitative analysis of posterior body tissue fluorescence intensity using ImageJ. * *p* < 0.05, ** *p* < 0.01, and *** *p* < 0.001 vs. G-fix. (**C**) Representative fluorescence images of fixed posterior body tissues after injection with Hoechst 33342, Fluoresbrite YG microspheres, or PI. Scale bar = 3.0 mm. (**D**) Quantitative comparison of fluorescence intensities from injected fluorescent materials versus background autofluorescence. * *p* < 0.05, ** *p* < 0.01, and *** *p* < 0.001 vs. G-fix.

Next, we evaluated autofluorescence following fixation with either 9% glyoxal (G-fix) or Davidson’s solution (D-fix). As shown in Figure 2A, both fixatives induced autofluorescence in the posterior body tissues, with varying intensities depending on the strain and wavelength. Under blue excitation, D-fix induced significantly higher autofluorescence than G-fix in all five strains. In contrast, under green and red excitation, G-fix induced significantly stronger autofluorescence than D-fix (Figure 2B).

To determine whether this background autofluorescence interferes with the detection of common fluorescent dyes, we injected Hoechst 33342, Fluoresbrite YG microspheres, and PI into the posterior body tissues. As shown in Figure 2C, all dyes produced significantly higher fluorescence signals than the background autofluorescence. ImageJ analysis revealed that in the blue channel, Hoechst 33342, exhibited > 200% higher fluorescence intensities than background in the G-fixed tissues across all strains (Y: 238.5%, R: 233.0%, Bu: 263.2%, Bk: 255.6%, and W: 266.0%) and were significantly higher than those in the D-fixed tissues (Y: 176.9%, R: 213.4%, Bu: 203.4%, Bk: 189.7%, and W: 219.7%). In the green and red channels, Fluoresbrite YG microspheres and PI exhibited 167.1–239.7% and 222.1–267.0% higher intensities than background in the G-fixed tissues, respectively. However, these values were significantly lower than those in the D-fixed tissues (Fluoresbrite YG: 270.6–333.1% and PI: 269.4–309.0%).

We also noted that autofluorescence varied by strain. Medaka with lighter body colors (Yokihi, red, and white) exhibited stronger autofluorescence, whereas darker-colored strains (blue and black) showed weaker signals.

### 3.2. Glyoxal- and Davidson-Fixed Medaka Brain Sections Exhibit Differential Autofluorescence Levels

**Figure 3:**
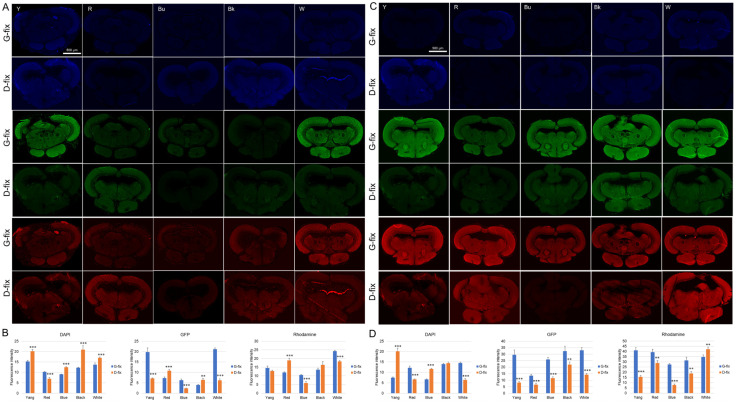
Glyoxal- and Davidson-fixed medaka brain sections exhibit different levels of autofluorescence. Brain tissues from five types of medaka fish (n = 10 per group) were fixed with either 9% glyoxal or Davidson’s solution, dehydrated, and embedded in paraffin: (**A**) representative fluorescence images of paraffin-embedded brain sections under various light sources showing fixative-induced autofluorescence (Scale bar = 500 μm); (**C**) representative images of deparaffinized brain sections stained only with secondary antibodies conjugated to Alexa Fluor 488 or 568 to assess background signal (Scale bar = 500 μm); (**B**,**D**) quantitative analysis of fluorescence intensities in brain tissues using ImageJ. ** *p* < 0.01 and *** *p* < 0.001 vs. G-fix.

To compare fixative-induced autofluorescence in brain tissue, paraffin-embedded brain sections (10 µm thick) were prepared from glyoxal- and Davidson-fixed samples of five medaka strains. Midbrain sections were deparaffinized and incubated with Alexa Fluor 488- or 568-conjugated secondary antibodies alone (without primary antibodies) to assess nonspecific autofluorescence. As shown in Figure 3A–D, G-fix resulted in significantly stronger green and red autofluorescence than D-fix across most strains, particularly in deparaffinized sections. In contrast, D-fix induced significantly stronger blue autofluorescence than G-fix in paraffin-embedded sections for most strains and in deparaffinized sections for the Yokihi and blue strains.

These findings indicate that G-fix induces higher background autofluorescence than D-fix in brain tissue, particularly in the green and red channels, commonly used for immunofluorescence. 

**Figure 4:**
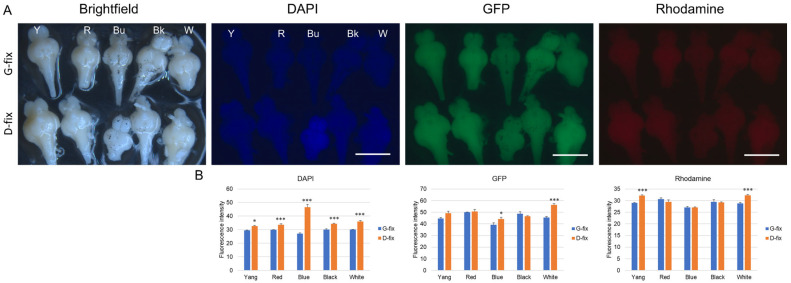
Autofluorescence of glyoxal- and Davidson-fixed medaka whole brain tissues. Five strains of medaka fish (n = 10 per group) were euthanized and fixed with either 9% glyoxal or Davidson’s solution, as detailed in Section 2 (Materials and Methods). Whole brain tissues were dissected from the skulls and observed using a fluorescence stereo microscope: (**A**) representative fluorescence images of the whole fixed brains under various light sources (Scale bar = 2.5 mm); (**B**) quantitative analysis of fluorescence intensities in whole brains using ImageJ. * *p* < 0.05 and *** *p* < 0.001 vs. G-fix.

To further assess whole-tissue fluorescence, we examined autofluorescence in intact brains fixed with G-fix and D-fix. As shown in Figure 4A,B, D-fix brain samples from all five medaka strains exhibited significantly stronger blue fluorescence compared to G-fix brains of the same strain, with the D-fix blue medaka showing the highest signal intensity. In contrast, there were no marked differences in green or red fluorescence between fixatives, except for slightly elevated green fluorescence in the blue and white strains and red fluorescence in the Yang and white strains.

Notably, autofluorescence levels observed in intact brains and paraffin-embedded brain sections did not consistently align with those observed after deparaffinization, particularly in the green and red channels. In contrast, autofluorescence patterns in posterior body tissue more closely matched those of deparaffinized brain sections. These results suggest that posterior body tissue may serve as a reliable and minimally invasive surrogate for assessing fixation-induced autofluorescence and for optimizing staining protocols prior to brain-tissue processing.

### 3.3. PGP9.5 Detection by Immunofluorescence but No Detectable NeuN, NCAM, or IHC Signals in Glyoxal- and Davidson-Fixed Medaka Brain Sections

To assess whether fixative-induced autofluorescence interferes with the detection of neuronal markers, we performed both IF and IHC for PGP9.5, NeuN, and NCAM in red medaka, the most cost-effective among the five strains.

**Figure 5:**
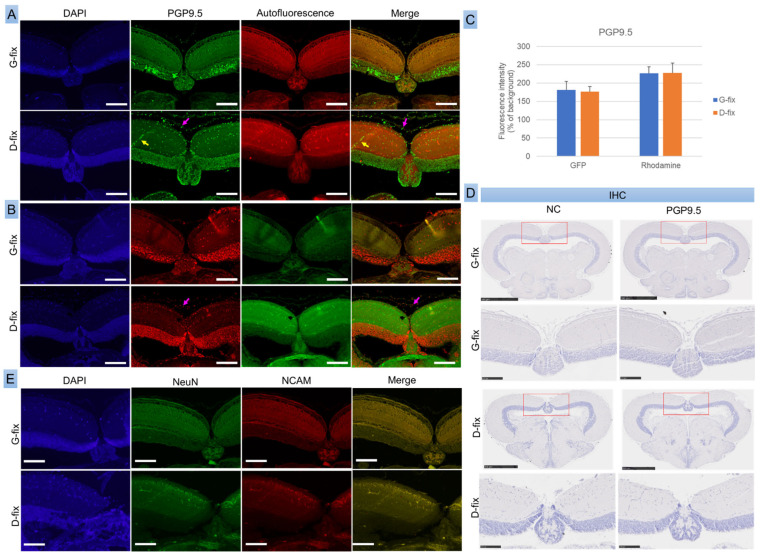
Glyoxal- and Davidson-fixed medaka brain sections exhibit comparable immunofluorescence signal intensities for the neuronal marker PGP9.5. Brain sections from red medaka fish (n = 10 per group) were stained with a primary antibody against the neuronal markers PGP9.5, NeuN, and NCAM, followed by secondary antibodies conjugated with either Alexa Fluor 488, Alexa Fluor 568, or HRP, as described in Section 2 (Materials and Methods). (**A**) Representative fluorescence images of brain sections stained with PGP9.5 and Alexa Fluor 488-conjugated secondary antibodies under different excitation wavelengths. (**B**) Representative images of sections stained with PGP9.5 and Alexa Fluor 568-conjugated secondary antibodies under the same conditions. Yellow arrows indicate red blood cells; pink arrows highlight connective tissue cells positively labeled for PGP9.5. Scale bar = 100 µm. (**C**) Quantitative analysis comparing PGP9.5 signal intensities to background autofluorescence using ImageJ. (**D**) HRP-stained brain sections visualized with the NanoZoomer S20 slide scanner. Red rectangles indicate the regions of the optic tectum shown in higher magnification. Scale bar = 500 µm (upper panels of G-fix and D-fix) or 100 µm (lower panels of G-fix and D-fix). (**E**) Representative fluorescence images of brain sections stained with NeuN, NCAM, and Alexa Fluor 488- or 568-conjugated secondary antibodies. Scale bar = 100 µm.

As shown in Figure 5A–C, PGP9.5 was clearly detected in both G-fix and D-fix brain sections using primary antibody and Alexa Fluor 488- or 568-conjugated secondary antibodies. The immunofluorescence signals were substantially stronger than background autofluorescence. Both fixatives yielded comparable signal intensities in the green and red channels. However, G-fix provided more specific labeling, restricted primarily to neuronal cells within the optic tectum, whereas D-fix produced broader staining, including non-neuronal elements such as red blood cells and connective tissue cells. 

IHC with HRP-conjugated secondary antibodies failed to detect PGP9.5 expression, likely due to insufficient sensitivity (Figure 5D). In contrast, the neuronal markers NeuN and NCAM were undetectable by both IF (Figure 5E) and IHC, probably because of species incompatibility, as these antibodies are not validated for teleosts—anti-NeuN reacts with human, mouse, and rat, while NCAM reacts only with human.

These findings suggest that PGP9.5 is a reliable neuronal marker for IF in medaka brain tissue, and G-fix offers greater labeling specificity than D-fix. 

### 3.4. Davidson-Fixed Medaka Brain Sections Exhibit Superior H&E Staining Quality Compared to Glyoxal-Fixed Sections

**Figure 6:**
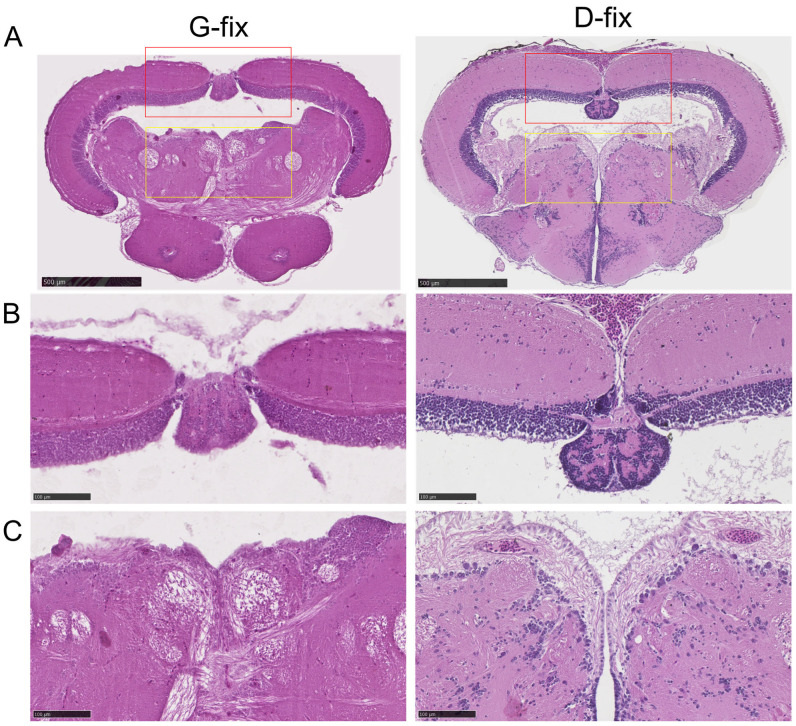
Davidson-fixed medaka brain sections demonstrate superior H&E staining quality compared to glyoxal-fixed sections. Brain sections from red medaka fish (n = 10 per group) were stained with H&E. Images were acquired using the NanoZoomer S20 slide scanner.(**A**) Typical image of a whole midbrain region. Scale bar = 500 µm. (**B**) Enlarged view of the optic tectum (red rectangles in the top panel). Scale bar = 100 µm. (**C**) Enlarged view of the central midbrain region (yellow rectangles in the top panel). Scale bar = 100 µm.

To evaluate the impact of fixation on histological staining, we compared H&E staining quality between glyoxal- and Davidson-fixed brain tissues. As shown in Figure 6A–C, D-fix midbrain sections exhibited excellent staining quality, with clear delineation of tissue architecture and high contrast of cellular components, particularly nuclei. In contrast, G-fix sections showed reduced contrast, with less distinct cellular features, except for neuronal fibers in the central midbrain region. These findings indicate that D-fix provides superior tissue preservation and staining quality for H&E in medaka brain sections compared to G-fix. 

## 4. Discussion

In this study, we systematically evaluated the effects of two widely used fixatives—glyoxal and Davidson’s solution—on autofluorescence, immunolabeling specificity, and histological quality in medaka, a well-established vertebrate model in neuroscience and developmental research. Our findings reveal key differences in fixative-induced autofluorescence and labeling performance that are critical for optimizing fluorescence-based imaging and antigen detection in fish models.

We first confirmed that fresh, unfixed posterior body tissues from five medaka strains showed no detectable autofluorescence under common excitation wavelengths. However, fixation with either glyoxal or Davidson’s solution induced varying levels of autofluorescence, particularly in the green and red channels frequently used in immunofluorescence microscopy. Notably, G-fix led to significantly higher autofluorescence in these channels, whereas D-fix induced stronger signals in the blue channel. These differences align with previous reports suggesting that aldehyde-based fixatives can cause cross-linking reactions that contribute to background fluorescence [17,18]. Interestingly, lighter-colored strains (Yokihi, red, and white) exhibited stronger autofluorescence than darker strains (blue and black), which may result from differences in pigmentation level and tissue optical properties such as light scattering and absorption. This finding suggests that pigmentation can influence autofluorescence levels in medaka and is consistent with previous reports in zebrafish and mice showing melanin-rich tissues exhibit reduced background fluorescence [19,20,21]. Such variation should be considered when designing fluorescence-based experiments in animal models. Importantly, we demonstrated that autofluorescence induced by either fixative remained significantly lower than the signals produced by fluorescent dyes, such as Hoechst 33342, Fluoresbrite YG microspheres, and PI, as well as by specific antibody-based labeling using Alexa Fluor 488 and 568. These findings suggest that, when properly controlled, fixative-induced autofluorescence does not substantially hinder the detection of biologically relevant fluorescence signals in medaka tissues.

Our study further revealed that G-fix produced more specific immunofluorescence labeling for the neuronal marker PGP9.5, with signal localization restricted to neuronal cells in the optic tectum. In contrast, D-fix resulted in broader staining, including non-neuronal elements such as red blood cells and stromal components. These findings suggest that glyoxal better preserves epitope integrity for immunofluorescence applications, likely due to its reduced protein cross-linking activity compared to formaldehyde-based fixatives [8]. This is particularly relevant for studies requiring precise cellular localization of disease-associated proteins or cellular markers in neurodegenerative, developmental, or toxicological models. Notably, NeuN and NCAM could not be detected by immunofluorescence even with G-fix, likely due to species incompatibility, as these antibodies are not validated for teleosts. This underscores the need for careful antibody selection in similar studies. Conversely, D-fix provided superior histological detail and H&E staining quality, especially in nuclear morphology. This makes D-fix highly suitable for histopathological assessments, including lesion scoring and tissue morphology evaluation in disease models.

Interestingly, autofluorescence patterns in posterior body tissue—rather than those in fixed whole brains or paraffin-embedded sections—closely resembled those in deparaffinized brain sections, supporting the use of posterior body samples as surrogate tissue for pre-screening fixation effects. This provides a practical, minimally invasive approach for optimizing fixation and staining protocols prior to processing brain or other internal tissues, particularly in studies involving rare, high-value, or transgenic fish lines. 

We observed that in medaka brain tissue, certain proteins—such as the neuronal marker PGP9.5—were detectable by IF but not by IHC, while others, including COX-2 and HIF-1α, were detectable by IHC but not by IF (data submitted). This suggests that antigen accessibility and detection sensitivity can vary with the method used. The absence of PGP9.5 detection by IHC, even with HRP amplification, may reflect the inherently lower sensitivity of chromogenic methods compared to fluorescence, especially when target proteins are weakly expressed or partially masked by fixation. In small fish brain tissues, chromogenic detection may also be limited by low signal intensity, reduced spatial resolution, and difficulty distinguishing weakly expressed or poorly preserved epitopes from background in densely packed neuronal regions. Conversely, COX-2 and HIF-1α may require the chromogenic conditions of IHC to maintain antigen stability or localization within fixed tissues [22]. These findings highlight the importance of carefully selecting both the fixative and detection method when designing immunolabeling protocols for medaka and other small model organisms. 

## 5. Conclusions

Taken together, our findings demonstrate that glyoxal and Davidson’s fixatives each have distinct advantages for medaka brain-tissue processing. G-fix offers higher specificity in immunofluorescence labeling, particularly for neuronal markers, while D-fix provides superior histological detail and lower background autofluorescence in the green and red channels. Importantly, fixative-induced autofluorescence does not significantly interfere with the detection of fluorophore-conjugated antibodies or fluorescent dyes when appropriate controls and imaging settings are applied. These results offer practical guidance for researchers working with small teleost models, highlighting that fixative choice can significantly influence imaging quality. Glyoxal fixation is well suited for studies requiring precise protein localization, while Davidson fixation is advantageous for structural and histopathological assessments.

Additionally, our use of 3D printed fish holders standardized euthanasia and dissection procedures, offering a simple and reproducible method to enhance consistency in small fish histology. By clarifying how fixation choice influences autofluorescence and antigen detectability, this study provides practical guidance for optimizing immunolabeling and histological protocols in teleost models. These findings support the refinement of fluorescence imaging techniques and reinforce the utility of medaka as a model organism in neurobiology, toxicology, and related biomedical research.

## Data Availability

The data that support the findings of this study are available within the article. Other data related to this study are available upon request from the corresponding author.

## Supplementary Materials

The following supporting information can be downloaded at: https://www.mdpi.com/article/10.3390/biomedicines13082002/s1, Supplemental File S1: fish matrix-1.
